# P-1074. Carbapenem-Resistant Acinetobacter baumannii infection in the hospitalized patients

**DOI:** 10.1093/ofid/ofaf695.1269

**Published:** 2026-01-11

**Authors:** nora Ranjitkar, Piyush Rajbhandari, Shreyashi Tuladhar, Rajni Lama, Janak Koirala

**Affiliations:** patan Academy of Health Sciences, Lilitpur, Bagmati, Nepal; Patan Academy of Health Sciences, Lalitpur, Bagmati, Nepal; Patan Academy of Health Sciences, Lalitpur, Bagmati, Nepal; Patan Academy of Health Sciences, Lalitpur, Bagmati, Nepal; Patan Academy of Health Sciences, Lalitpur, Bagmati, Nepal

## Abstract

**Background:**

Globally *Acinetobacter baumannii* is the fifth leading cause of death among the antimicrobial resistant (AMR) organisms. World Health Organization (WHO) has listed Carbapenem-Resistant *A baumannii* (CRAB) in the critical priority list. There is a paucity of data to support the choice of treatment of CRAB infection with combination antibiotics in Nepal and South Asia. We describe incidence of CRAB and explore the outcomes of treatment with combination antibiotics for CRAB infections.

**Methods:**

This is an ongoing prospective study at Patan Academy of Health Sciences, Nepal. All patients above the age of 18 years meeting predefined clinical criteria of CRAB infections were identified and enrolled in the study. Susceptibility tests were performed using Kirby-Bauer and MIC methods. Extensively-drug resistance (XDR) was defined as resistance to all antibiotics except colistin and doxycycline. Patients received one of the three combination treatments: (A) Colistin plus Meropenem; (B) Colistin plus high dose Ampicillin-Sulbactam; and (C) Colistin plus high-dose Ampicillin-Sulbactam plus Minocycline. After enrollment, patients were followed daily in hospital, and by telephonic conversation on days 7 and 28 after enrollment, if discharged.

**Results:**

Data was collected from Dec 1, 2024 to March 31, 2025. Of the 128 samples positive for CRAB, 120 isolates were XDR. All isolates tested were susceptible to Colistin but only 53 (41.4%) were susceptible to Doxycycline. Of the 13 patients who received combination treatments, 4 patients received combination (A), 5 received (B), and 4 received (C). All 13 patients had hospital acquired infections- 6 hospital-acquired pneumonia (HAP), 6 ventilator-associated pneumonia (VAP), and 1 central-line exit site infection. Clinical cure was achieved by 12 patients (92%). Total hospital-stay ranged from 17-60 days. Total ICU stay after randomization ranged from days 2-22 (median 7 days). At day seven, mortality was observed in 2 patients, both in group C. At 28 days, mortality was observed in 5 patients (1 in A, 1 in B and 3 in C).

**Conclusion:**

Although clinical cure rate was high with combination antibiotics, hospital-acquired infections with CRAB had the high rates of mortality at days 7 and 28. It was also associated with the longer ICU and hospital stay.

**Disclosures:**

All Authors: No reported disclosures

